# Research conducted on Caribbean women's perceived human immunodeficiency virus risks: A narrative review and methodological critique

**DOI:** 10.1080/21642850.2014.905209

**Published:** 2014-04-23

**Authors:** Su-Anne Robyn Charlery

**Affiliations:** ^a^ Department of Health Promotion and Behavior, University of Georgia, Athens, GA, USA

**Keywords:** HIV, Caribbean, women, perceived risk

## Abstract

*Background*: Caribbean women have the highest human immunodeficiency virus (HIV) infection rates among women in the Americas; however, their self-assessment of HIV risk is alarmingly low. This reflects a low perceived risk for HIV. English-speaking Caribbean countries are typically understudied in this area. It is important for health researchers and practitioners to understand the underlying perceptions of women who are now driving this epidemic. This review discusses and critiques the published literature that examines Caribbean women's perceived HIV risks. *Methods*: Medline, PsycINFO, Global Health, Women's Studies International, and Academic Search Complete databases were searched using various combinations of the following keywords: *Caribbean, women, HIV, STD, AIDS, risk, perceived risk, risk perception, and sex.* Searches were restricted to English. A total of 69 peer-reviewed studies were obtained from the initial 239 records. The reviewer screened the peer-reviewed articles and excluded 50 studies that did not directly assess perceived HIV risks in Caribbean women. An additional 12 studies were excluded based on the following exclusion criteria: an undetermined proportion or more than 50% of the sample consisted of pregnant women, sex workers, drug users, Latinas, and/or people living with HIV/AIDS. *Results*: Seven studies on perceived HIV risk in Caribbean women were reviewed. Jamaican women were the most represented ethnic demographic (43%). All studies assessed perceived risk as a subset of HIV psychosocial factors, sexual-risk behaviors, HIV knowledge, attitudes, and beliefs. Four studies used cross-sectional research design and two studies used qualitative methodology. Only one study described items used to measure perceived risk. General findings indicate overall perceptions of invulnerability among Caribbean women, despite high sexual-risk behaviors. *Conclusions*: Published studies that specifically assess Caribbean women's HIV risk perceptions are currently lacking. Qualitative research is needed to further evaluate and explore perceived risks. This will better inform practical strategies that can enable women to discern between their perceived and actual risks, and invariably reduce sexual risk-taking behaviors.

## Introduction

The Caribbean has the second highest incidence of human immunodeficiency virus (HIV) and acquired immune deficiency syndrome (AIDS) in the world after sub-Saharan Africa, with a prevalence rate of approximately 2% among the population aged 15–49 (UNAIDS, 2013). This is considerably higher than the 0.8% prevalence estimated for the world population of the same age group. In this region, approximately 65% of HIV/AIDS cases have been attributed to heterosexual transmission – therefore, this is the most common route of infection for both men and women (Figueroa, 2008; UNAIDS, 2010c). HIV transmission is propagated by inconsistent or non-existent condom use among sexual partners, which is highly reflected by research conducted in the Caribbean. For instance, on the island of Saint Lucia, only 45% of people who reported having sexual intercourse with non-regular partners in the past 12 months used condoms during their last sexual activity (Allen, Simon, Edwards, & Simeon, 2010). Early sexual initiation, another risk factor for HIV/AIDS, is also very common in the Caribbean region among adolescents. Among adolescents who reported having had sex in the Caribbean Youth Health Survey that surveyed half of the Anglophone Caribbean countries (Antigua, Bahamas, Barbados, British Virgin Islands, Dominica, Grenada, Guyana, Jamaica, and Saint Lucia), almost two-thirds of them reported sexual initiation before the age of 13 (Halcón et al., 2003).

Caribbean males generally exhibit a trend of outnumbering their female counterparts with regard to their number of sexual partners. Research in the Caribbean indicates that males are up to three times more likely than females to have had anywhere from two to upwards of five more sexual partners than females (Halcón et al., 2003; Harris-Hastick & Modeste-Curwen, 2001; Kurtz, Douglas, & Lugo, 2005; Maharaj, Nunes, & Renwick, 2009; O'Toole, McConkey, Casson, Goetz-Goldberg, & Yazdani, 2007). According to UNAIDS (2010c), women and young girls have become the most vulnerable and most exposed group to the current HIV/AIDS epidemic in this region. Approximately, 53% of all HIV cases reported in the Caribbean are in women, making this the highest rate of HIV infection reported among women in the Americas (UNAIDS, 2007). Studies conducted in developing African and Asian countries (Zaire, Tanzania, Rwanda, and Thailand) and in American minority populations (African–Americans, Hispanics, and Native Americans) indicate that major factors influencing the impact of HIV/AIDS on females are their economic dependence on men, unequivocal societal gender roles, and their poor sexual and condom negotiation skills (Wingood & DiClemente, 2000). In these groups of women, high levels of poverty and economic dependency increase their vulnerability to contracting sexually transmitted infections (STIs) and HIV. These factors may very well apply to the Caribbean region but each island has its own ‘distinctive personality and flavor’ thus contributing factors driving this epidemic in one island may not necessarily be mirrored in another.

A report on research conducted in St. Kitts, St. Vincent, and Barbados indicates that self-assessment of HIV risk is very low among the females surveyed, as approximately 50% of those who have unprotected sex still do not consider themselves at risk of contracting STIs or HIV (Avant Garde Media, 2008). This report purports that the sample of females possesses a low perceived risk for HIV and its personal relevance to their lives. The main idea of HIV perceived risk in psychosocial and health theoretical models such as the health belief model (HBM) is that as persons recognize that their behaviors increase their risk of HIV infection, they are more likely to adopt healthier behavior changes to reduce this risk than others who do not recognize their risk (Rosenstock, Strecher, & Becker, 1994). Perceived risk is often posited as a necessary change for behavior and is a central construct in numerous health behavior models. For example, the HBM, the theory of reasoned action, the Information, Motivation, and Behavioral Skills model, and the AIDS Risk Reduction Model, all share perceived risk as a central concept.

Latin American and Caribbean countries are often grouped together for HIV surveillance; however, there are very stark differences both between and within the Latin American region and Caribbean region such as culture, political climate, and economic status (Calleja et al., 2002; Huedo-Medina et al., 2010). Most relevant to this topic are the differences in the nature of the HIV/AIDS epidemic in many Latin American versus Caribbean countries such as high HIV transmission driven via injection drug use in Brazil and female sex workers in the Dominican Republic versus generalized epidemics driven by heterosexual behavior in Bahamas and Jamaica (Calleja et al., 2002). Given the unique sociocultural makeup of these countries, it is beneficial to explore HIV-related factors prevalent within the Caribbean region as its own entity.

Prior to designing and administering HIV prevention interventions in the Caribbean community, it is important for health professionals to first gain an in-depth understanding of the perceptions, beliefs, and attitudes of Caribbean females who engage in the heterosexual activity that has been noted to drive this epidemic (UNAIDS, 2010b). This is instrumental in order to develop successful prevention strategies that can reduce HIV transmission in this vulnerable population. This paper reviews and examines the methodological quality and measured outcomes of peer-reviewed studies published on HIV-risk perceptions of Caribbean females, as well as the factors that contribute to their perceived risks.

## Methods

### Search strategy

A search of the published literature related to Caribbean women's HIV-risk perceptions and HIV-related beliefs was conducted using the following electronic databases: PsycINFO, MEDLINE, Global Health, Women's Studies international and Academic Search Complete. A combination of the following search terms were utilized: Caribbean, women, HIV, human immu* virus, AIDS, acquired immu* syndrome, STD, STI, risk, perceived risk, risk perception, and sex. Searches were restricted to English language journals.

### Inclusion and exclusion criteria

For this review, studies had to meet several inclusion criteria prior to being selected. Studies conducted in the Caribbean that were available by October 2013 were included in this study if they were (1) original published research based on primary or secondary data – not including meta-analyses or other reviews; (2) full text articles published in English; and (3) direct assessment of HIV risk/perceived risks in a sample including a significant proportion of Caribbean women. Additionally, further inclusion criteria were that the studies would not target populations primarily consisting of pregnant women, sex workers, drug users, people living with HIV, and Latinas as a significant portion of the studies sample (i.e. more than 50%).

This study reviews published research that targets sexually active heterosexual women, who were not specifically recruited for being part of an identifiable group that considerably elevates or lowers their risk for HIV (i.e. sex workers, drug users, people living with HIV, and pregnant women, respectively). Pregnant women have circumstances that set them apart from non-pregnant women. They are thought to experience a lower risk of HIV acquisition during pregnancy and their HIV status is more likely to be concordant with their current sexual partner than non-pregnant women (Marston et al., 2013). Studies including a majority proportion of Latinas (women from Central America, South America, and Spanish-speaking Caribbean countries) are also omitted due to the differences that exist in the sociocultural drivers and nature of the HIV/AIDS epidemic in Latin America and the English-speaking Caribbean (Calleja et al., 2002; Huedo-Medina et al., 2010).

### Study selection

**Figure 1.**
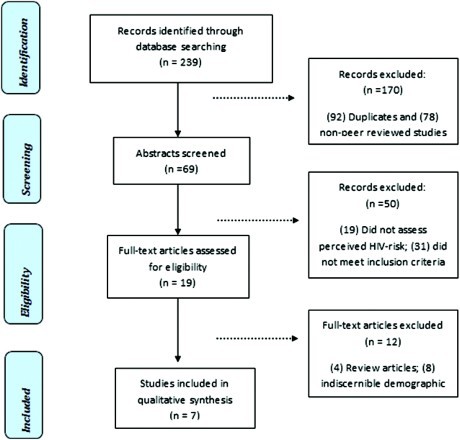
Selection process for review articles.

The literature search yielded 239 studies, of which 92 were duplicates and 78 were not peer-reviewed studies. The reviewer screened the abstracts of the remaining 69 peer-reviewed studies based on the inclusion criteria listed above to determine relevance for inclusion in this study. Nineteen studies did not directly assess perceived HIV risks in Caribbean women as a component of their inquiry process and were excluded at this stage. An additional 31 studies were excluded because they did not meet the inclusion criteria: more than half of the sample consisted of pregnant women, sex workers, drug users, Latinas, and/or people living with HIV/AIDS. Nineteen articles were considered to be relevant and were examined in depth. Twelve studies were excluded at this stage, which comprised four review articles and eight studies with an indiscernible or unspecified proportion of Caribbean women. Finally, a total of seven studies were considered relevant and met all inclusion criteria for this study (Figure 1).

### Study appraisal

In order to examine the quality of the studies, the following areas were investigated: appropriate study design as it relates to the research question(s) or hypotheses, quality of sample size and participant demographics, appropriate instrument measures, method of administration, and the impact of the results. Appropriate study design was defined as reviewed studies having a clear description and methodologically sound justification for the design used. Studies must have clearly defined the population and justified how participants were recruited and selected. Additionally, a clear theoretical framework should inform the research design and components. Quality of instrument measures was evaluated based on the clear identification of scale validation, description, and evaluation of items used to measure constructs, and appropriateness for the target populations in studies reviewed. The quality of method of administration was examined based on disclosure of the setting for program or questionnaire administration and the length of time allotted for administration. Finally, the impacts of the results were examined by the positive or negative association of the HIV perceived risks to measured actual or intended risky behavior. Additionally, results were considered to have a positive impact if any of the outcomes reduced the risk of the participant to engage or report risky sexual behavior. The perceived HIV risks of women reported in each study was compared and contrasted for this purpose. A summary of all studies included in this review can be found in Table 1.

**Table 1.** Summary of critiqued articles.

Author (date)	Purpose	Sample description and research setting	Research design
(1) Baird, Yearwood, and Perrino (2007)	To investigate effective methods to promote safer sex behavior and to reduce HIV risk	50 females; ages 15–21; Trinidad and Tobago	Experimental: pre–post intervention/quasi-experimental
(2) Campbell-Stennett, Holder-Nevins, McCawBinns, and Eldemire-Shearer (2009)	To identify factors influencing the stage of change in regard to HIV testing in women	372 women; ages 16–45; sexually active; self-reported HIV negative; Westmoreland, Jamaica	Cross-sectional
(3) Elisburg (2002)	To explore the gap between knowledge and attitudes of HIV/AIDS and contraception and behavior change	90 women; ages 18–46; Portsmouth (Health district), Dominica	Qualitative
(4) Gillespie-Johnson (2008)	To explore cultural factors and health beliefs influencing HIV/AIDS prevention behavior in young, single, heterosexual, recent-immigrant Jamaican women	20 single, heterosexual, Jamaican women immigrants to USA <12 years ago; sexually active; ages 18–30; Miami-Fort Lauderdale metropolitan area	Qualitative (Heidegger's hermeneutic interpretive phenomenological)
(5) Malow, Cassagnol, McMahon, Jennings, and Roatta (2000)	To describe the prevalence of HIV-risk behavior among low-income Haitian women, identify psychosocial variables predictive of HIV-risk behavior, and provide formative data to guide development of interventions for this population	101 Haitian women; ages 18+; ‘Little Haiti’ Miami, FL	Cross-sectional
(6) Reynolds, Beauvais, Lugina, Gmach, and Thomsen (2010)	To investigate whether youth who use voluntary counseling and testing (VCT) services report risky behavior for HIV and unintended pregnancy, and whether youth reporting risky behaviors consider themselves to be at risk for HIV. (Focus on HIV testing and reproductive health.)	401 16–24-year olds in Tanzania; 366 15–24-year olds in Haiti (169 female); VCT centers in Port-au-Prince, Haiti Dar es Salaam, Tanzania	Cross-sectional
(7) Warren (1999)	To examine whether geography influences HIV/AIDS awareness, sources of info, knowledge of and attitudes toward HIV/AIDS	913 girls, ages 14–17; (urban versus rural) Kingston, Jamaica	Cross-sectional

## Results

### Sample of studies

In this paper, seven studies meeting the overall inclusion criteria for this study were summarized then systematically reviewed based on the following categories: sample size and participants, research design, theoretical application procedures, use of theory, measures, and outcomes.

### Sample size and participants

Six studies exclusively recruited females who were identified as being from the Caribbean, whereas one study recruited both males and females, and included participants from Tanzania in addition to its Haitian participants (Reynolds et al., 2010). Only two studies were conducted outside of the Caribbean, and both took place in Miami, Florida. The researchers recruited women living in the area that were identified as being of Caribbean descent. One study recruited Haitians and the other specifically targeted Jamaicans (Gillespie-Johnson, 2008; Malow et al., 2000). Another study used both pregnant (*n* = 173) and non-pregnant (*n* = 199) Jamaican women in its sample (Campbell-Stennett et al., 2009). The five studies conducted in the Caribbean occurred in Trinidad and Tobago, Dominica, Jamaica, and Haiti. Sample sizes ranged from 20 to 90 participants for the qualitative studies and from 101 to 913 for the quantitative studies. Participants' ages ranged from 14 to 46 with almost half of the studies exclusively including participants younger than 24 years old (Baird et al., 2007; Reynolds et al., 2010; Warren, 1999).

### Research design

Four studies utilized a cross-sectional research design (Campbell-Stennett et al., 2009; Malow et al., 2000; Reynolds et al., 2010; Warren, 1999), two used qualitative designs (Elisburg, 2002; Gillespie-Johnson, 2008), and one was an experimental pre–post intervention design (Baird et al., 2007).

### Use of theory

Only two studies used theoretical frameworks to guide the research. One used the trans-theoretical model to guide the development of the instrument used (Campbell-Stennett et al., 2009) and the other used the HBM as a framework to guide the process of inquiry (Gillespie-Johnson, 2008).

### Instruments to assess HIV-risk perceptions

A series of instruments were used to assess HIV-risk perceptions and other constructs in the studies reviewed including adaptations or modifications of previously validated scales, newly created scales, and in-depth interviews.

Two studies outlined their adaptation of validated instruments or scales as measures to assess the constructs: Baird et al. (2007) modified the Be Proud! Be Responsible! (BPBR) HIV prevention program instrument used by Center for Disease Control to help assess sexual-risk behavior of African–American youth and Malow et al. (2000) adapted the previously validated AARM – Questionnaire Revised (AARM-QR). Elisburg (2002) modified and reorganized questions from focus group to interview format retrieved from the Caribbean Epidemiology Center (CAREC, 1994) and Family Health International (Ulin, Cayemittes, & Metellus, 1993) projects for use in her study. The instrument was culturally tailored and modified for the target population of Dominican women. Two studies created instruments that were guided by theory (Campbell-Stennett et al., 2009; Gillespie-Johnson, 2008) and the remaining two did not report their instrument development process (Reynolds et al., 2010; Warren, 1999).

#### Measurement items

Likert-type scales were utilized by two of the quantitative studies (Campbell-Stennett et al., 2009; Warren, 1999). Malow et al. (2000) and Reynolds et al. (2010) did not describe items used to measure constructs, neither did they report the quantity of items used. Baird et al. (2007) also did not describe the four items used to measure perceived risk but mentions that the questionnaire's items had an overall internal consistency of 0.05 and test–retest reliability. The only example of a measurement item provided was from Campbell-Stennett et al.’s (2009) study which states ‘Do you think you are at risk of contracting HIV?’ The responses ‘disagree’ and ‘strongly disagree’ were coded as *no risk*, and the responses ‘agree’ or ‘strongly agree’ were coded as *some risk*. In the qualitative studies, Elisburg (2002) used a 26-item structured questionnaire interview with questions about HIV knowledge, beliefs, and attitudes to elicit responses about HIV perceived risks. Gillespie-Johnson (2008) used a nine-item researcher administered questionnaire using one global question for each of their research question and for each of the six constructs of the HBM, which includes perceived susceptibility.

#### Method of administration

Most of the questionnaires were administered in clinics and lasted on average 30 minutes. Baird et al. (2007) administered the questionnaire pre- and post-intervention but did not specify what location or setting was used to do so. The questionnaire lasted 30–45 minutes. Warren (1999) self-administered questionnaires occurred in students' naturalistic classroom setting. Questionnaires administered in clinics varied by method of administration as one was an interviewer-assisted questionnaire administered in antenatal or child health clinics (Campbell-Stennett et al., 2009), and another was a confidential self-administered questionnaire administered in inner-city medical clinic for obstetrical and gynecological care (Malow et al., 2000). One study's qualitative in-person in-depth interviews lasted about 20 minutes (Elisburg, 2002) and Gillespie-Johnson (2008) did not describe the length of time for their qualitative interviews. Neither of the qualitative studies discussed the setting in which interviews occurred.

### Outcomes

The primary outcomes of interest evaluated for these studies are the HIV-risk perceptions of Caribbean females, and the factors contributing to these perceptions. The only experimental study found that perceived HIV risks significantly increased post-intervention in the experimental group (Baird et al., 2007). The authors determined that the BPBR cognitive-behavioral intervention may be effective in changing the HIV-risk perceptions of females, as well as sexual attitudes and feelings about themselves. Overall, the cross-sectional studies found that the majority of females reported a low to moderate personal perception of HIV risk. Campbell-Stennett et al. (2009) indicated that 60% of non-pregnant women in their sample perceived themselves as having no risk of becoming HIV infected although they reported receiving significantly more HIV tests than the pregnant women in the sample.

Reynolds et al. (2010) found that a considerably high proportion (66%) of the Haitian women reported behaviors that put them at higher risk for HIV, yet 78% of them thought they were at *low* or *no* risk of getting HIV. In examining the geographical variations of HIV-risk behavior, Warren (1999) determined that geography (living in urban versus rural areas) had a significant effect on young Jamaican girls' HIV-risk perceptions. Those living in more urbanized parts of the capital city of Kingston were more likely to perceive their risk as high than those who lived in the more rural areas of the city. Malow et al. (2000) report that despite 70% of their sample not using condoms during sexual activity in the past 30 days, they reported moderate perceived susceptibility to getting HIV. There was notably no indicator used to measure the effects of acculturation in this study, but this sample of Haitian-born women currently living in Miami reported very similar results to the Haitian women in the study conducted by Reynolds et al. (2010).

The qualitative studies indicated similar results as the quantitative studies with 59% of participants stating that they did not consider themselves at risk although 25% of them were uncertain about their partner's fidelity (Elisburg, 2002). It was interesting to note in the study conducted by Gillespie-Johnson (2008) that many of its Jamaican-born participants revealed having never given thought about their actual HIV risk, but realized during the study that they might actually be at risk. This study also did not account for acculturation effects of the Jamaican-born women in Miami, but shared similar findings as the other studies regarding its participants' HIV perceived risk. Almost all the studies report that the women had moderate to excellent knowledge of HIV, its transmission, and for the most part its prevention; however, there were some reported misconceptions about the efficacy of condom use (Malow et al., 2000) as well as cultural beliefs and practices that prevent protective sexual behavior (Gillespie-Johnson, 2008). Additionally, the frequency and acceptance of male infidelity with multiple sexual partners were found to be a common trend in both qualitative studies.

## Discussion

This paper provided a critique of seven studies that assessed HIV-risk perceptions of Caribbean and Caribbean born females. Overall, all the reviewed literature assessed HIV-risk perceptions of Caribbean females from a psychosocial perspective, i.e. evaluating sexual-risk behaviors, and/or HIV knowledge, and attitudes and beliefs. Ultimately, in light of the limited number of published literature available for review on this topic, it was apparent that there is a lack of research that specifically assesses HIV-risk perceptions of Caribbean females both in and outside the Caribbean.

The majority (4) of the reviewed studies utilized a cross-sectional design (Campbell-Stennett et al., 2009; Malow et al., 2000; Reynolds et al., 2010; Warren, 1999) and all used primary data. Although cross-sectional studies are helpful to determine associations and relationships among variables, they are also limited in their ability to determine the direction of causal relationships between variables, and also to adequately predict future behavior of participants. Thus, the reviewer deemed cross-section designs as suitable choices for the scope of these studies.

Experimental and quasi-experimental studies are typically considered as a strong research design among health researchers. However, there were a few issues with the methodological quality of the one quasi-experimental study reviewed (Baird et al., 2007). First, the sample was recruited via targeted sampling with flyers advertised in Trinidad and Tobago's high schools and local communities rather than using random sampling. This limits the external validity of the study's results as it is not generalizable to all the sexually active adolescents in Trinidad. This study reported statistically significant improvements when comparing the experimental group's pre- and post-test scores for feelings about sex, sexual attitudes, and perceived risks. However, the reviewer noted that this study reported between-group differences for only one out of six dependent variables (sexual attitudes). Therefore, it is possible that other factors could have contributed to the improvement of scores in both experimental and control groups. Additionally, there was no long-term follow-up with the participants so it is unknown if the intervention has had any lasting effects on perceived risk for HIV. Finally, this study used a small sample size of 100 (roughly 50 in each condition). By using such a small sample size, the study faces the threat of having a very low statistical power. Due to their small sample size, their results are potentially less accurate and reliable than studies done with a larger sample.

The interview is the most commonly used methodological tool for data collection in qualitative research (Enosh & Buchbinder, 2005; Glesne, 2011), so it seems plausible that the two qualitative studies reviewed utilized in-depth interviews to elicit responses from their participants (Elisburg, 2002; Gillespie-Johnson, 2008). Before a researcher can gather sensitive information from participants about their personal HIV-risk beliefs, it is important to build a rapport. According to Roulston (2010), when conducting research on a sensitive topic such as this one, researchers should assume a ‘romantic conception of the interview process’ which allows them to build a foundation of trust with the interviewees. This, in turn will encourage the interviewee to feel comfortable enough to reveal true feelings, thoughts, beliefs, and emotions during the conversation on intimate topics (Roulston, 2010, p. 56). This rapport-building practice was not described or implied in either of the qualitative studies reviewed, which may inherently have impacted the nature of the information the participants divulged to the researchers.

Elisburg (2002) used structured interviews in her study, whereas Gillespie-Johnson (2008) used semi-structured interviews. Within qualitative studies, using a highly structured interview with pre-established questions does not fully allow the data to guide the process of interviewing and can create a more rigid atmosphere (Rubin & Rubin, 2005). When compared to semi-structured interviews, highly structured interviews have the advantage of the reliability and consistency of having respondents answer the same questions. Posing identical questions to all participants also makes it easier to code resulting data (Creswell, 2007). However, highly structured interviews can deny the interviewer the opportunity to add, remove, or change questions as the interview progresses, which may be very useful to provide thick rich data (Roulston, 2010; Rubin & Rubin, 2005). Additionally, the stipulated interviewing time of 20 minutes in the study conducted by Elisburg (2002) is shorter than average (45 minutes to 1 hour) for studies of this nature (Shank, 2006), and potentially negatively affects the quality of data retrieved.

The steps taken to ensure reliability (credibility) and validity (transferability) of results in qualitative research is also critical to the quality of the study. Although the use of qualitative methods fit the scope of the study conducted by Elisburg (2002), they provided very limited description about the details of the research process which would inherently reduce the methodological rigor of the study in terms of credibility and transferability. On the other hand, Gillespie-Johnson (2008) provided a detailed outline of their data collection and analytic process similar to that of an audit trail, as well as their theoretical perspectives and assumptions to ensure credibility and transferability. They also highlighted their use of modified member checking. Member checking is a technique that increases rigor in qualitative research by the researcher cross-checking with participants for emerging themes and results at any stage of the analytic and interpretive process (Glesne, 2011). This process makes it easier for researchers to evaluate the quality of the study and findings in terms of its credibility (Krefting, 1991).

The studies reviewed most often used self-selected convenience samples recruited from clinics or schools. Therefore, the overall results of these studies are not generalizable to the general population of females within the Caribbean nationalities represented in the studies (i.e. Jamaican, Haitian, Trinidadian, and Dominican). The most represented Caribbean nationality focused on in these studies was Jamaican (three studies), followed by Haitian (two studies). This is not surprising, as Jamaica and Haiti, the largest English- and French-speaking islands in the Caribbean, respectively, receive considerably more donor support for HIV/AIDS-based research as opposed to the smaller islands such as Trinidad and Dominica (UNAIDS, 2013) – where the remaining two studies were conducted.

Jamaica currently has an HIV/AIDS prevalence rate of 1.7% and Haiti has a slightly higher rate of 1.9% (UNAIDS, 2010a). At a first glance, it may seem fitting to target people from these countries for HIV/AIDS research but there are countless other Caribbean nations currently suffering from this epidemic that remain understudied. For instance, Trinidad has an infection rate of 1.5% which although not as high as Jamaica's and Haiti's is still a cause for concern; however, it is not as highly represented in the literature. Smaller and less-researched islands, such as Dominica and Saint Lucia, have not received much generated interest from global donors and stakeholders thus there have been no large sero-prevalence studies (Pilgrim & Blum, 2012). Conducting sero-prevalence studies is necessary to provide good indicator of HIV rates in the general population.

Theoretical perspectives or paradigms were generally not highlighted within the reviewed studies. In particular, only two studies clearly identified the use of a theoretical framework to guide their research (Campbell-Stennett et al., 2009; Gillespie-Johnson, 2008), and reportedly used traditional psychosocial frameworks, namely the Trans-theoretical Model and the HBM. Theory, research, and practice are part of an iterative process; theory should be informed by practice and good practice should be grounded in theory (Glanz, Rimer, & Viswanath, 2008). Health researchers can use theory and its applications to guide their selection of constructs that are hypothesized to influence health behavior. The lack of theory used in these studies, particularly in guiding instrument development, weakens the overall quality of the study. Traditionally, psychological and behavioral theories were thought to be the ideal foundation of any social–behavioral study, particularly when trying to assess health behavior and beliefs. As time and health-behavioral research progressed, emergent theories such as the theory of gender and power were developed to further expand on psycho-behavioral theories and account for cultural influences (Wingood & DiClemente, 2000). Such emergent theories would be considered particularly useful for exploring contextual issues such as perceived and actual HIV risk in women, as researchers have critiqued the use of traditional theoretical applications in assessing issues heavily influenced by cultural differences (Albarracin, Fishbein, Johson, & Muellerleile, 2001; Jacobs, 2008; Rosenthal & Levy, 2010).

Additionally, it is important for ontological and epistemological perspectives to be discussed as it influences the analytic strategy of the research. Interview development should also align with the researcher's theoretical perspective and epistemology. These perspectives are essentially embedded in social research and provide a lens which shapes how the researcher views phenomena under study (Pascale, 2011). Gillespie-Johnson (2008) explicitly described their paradigm as hermeneutic interpretive phenomenological and was the only qualitative study to do so.

Another key critique of all seven studies is the lack of description provided about the construct items used to measure perceived risk in the studies. Most likely, this lack of description is related to the fact that perceived HIV risk was not the primary outcome of interest for any of the studies, but rather, the authors intended to assess this construct within the context of beliefs or psychosocial factors that contribute to HIV risk. Only a limited number of instruments were previously validated and/or pilot tested in representative samples of the population (Baird et al., 2007; Malow et al., 2000; Warren, 1999). Neglecting to validate and pilot test instruments has potentially negative implications on the suitability and construct validity of the instrument for the target populations.

Only one study provided a concrete example of a measurement item for perceived risk, which used a Likert-type scale to measure the response (Campbell-Stennett et al., 2009). Using Likert-type scales for this type of research is potentially problematic as it requires considerable decision-making on the participant's part to select a response, and the participants may either intentionally or unintentionally give incorrect answers – which could partly be due to feelings of social desirability (Kothari, 2004, p. 86). The issue of social desirability is crucial to instrument development and measures used, particularly when administering an interview-assisted questionnaire. When working with Caribbean populations such as Haitians who are predominantly Catholic and deeply rooted in their cultural and religious traditions (Inciardi, Syvertsen, & Surratt, 2005), researchers should take into consideration that their responses may be based on what they believe would make them appear better to the researcher. It is evidenced by researchers that condom use is likely to be discouraged by several religious and cultural norms among these populations (Braithwaite & Thomas, 2001); however, none of the studies mentioned accounting for social desirability in their measures.

All the studies reported fairly similar findings for the HIV-risk perceptions of their participants. Although the women and girls expressed much knowledge about HIV/AIDS and its transmission, they were sometimes deficient in their knowledge of HIV prevention practices. Some studies described the common misconceptions around condom efficacy, which was not surprising due to the deleterious effects of mixed messaging that occurs in many developing countries – condoms are advertised as the only effective mechanism to protect oneself from HIV during sexual intercourse, but at the same time, it is emphasized that condom use is not 100% effective (Figueroa, 2008). Overall, females generally held low to moderate perceptions of their risk for HIV, despite participating in risky sexual behaviors. This phenomenon reflects an overall perception of invulnerability among the Caribbean women participating in those studies. Despite their awareness of the severity of HIV and how it is transmitted, they continue to practice risky sexual behaviors under the illusion that they are not vulnerable to contracting HIV (Camara, 2006; Kibombo, Neema, & Ahmed, 2007; Panchanadeswaran et al., 2007). There are also cultural factors associated with this phenomenon such as the general acceptance of male infidelity, gender societal roles, religious and cultural practices, and traditions concerning condom use (Abel & Chambers, 2004; Fishbein, Middlestadt, & Trafimow, 1993).

## Conclusion

Based on the outcomes reported by the studies reviewed, HIV-risk perception of Caribbean females is best described as moderate to low, when in reality their actual HIV-risk behavior is reported as moderate to high. Factors associated with these perceptions include geography (urban or rural place of residence), cultural beliefs and traditions, limited and/or inconsistent male and/or female condom use, male partner infidelity, and misconceptions about HIV prevention methods such as condom efficacy.

Methodological issues in both quantitative and qualitative studies weakened the studies' findings and the quality of the research. Conclusions about HIV-risk perceptions drawn by the quantitative studies should be taken with caution due to limited description of the measures used to assess the strength of this construct and associated variables. Qualitative research indicated stronger potential to understand the contributing factors of females' HIV-risk perceptions when semi-structured or informal interviews guided by theoretical frameworks are utilized.

The limited literature available for this review suggests that the English-speaking Caribbean is understudied in the field of HIV/AIDS prevention research relative to the Spanish- or French-speaking territories such as Haiti and the Dominican Republic. This region has a predominant Black ethnic population, and thus this paper can contribute to the existing research on HIV risk and prevention on women of the African diaspora. Future research should focus on expanding the range of HIV-related studies to smaller understudied islands of the English-speaking Caribbean where the AIDS epidemic continues to take its toll. Additionally, the concept of perceived risk should be further investigated in Caribbean women to better inform strategies that can enable them to recognize their actual risks and subsequently reduce behaviors that essentially put them at risk of contracting HIV/AIDS.
